# Effects of a work schedule with abated quick returns on insomnia, sleepiness, and work-related fatigue: results from a large-scale cluster randomized controlled trial

**DOI:** 10.1093/sleep/zsae086

**Published:** 2024-04-06

**Authors:** Ingebjørg Louise Rockwell Djupedal, Anette Harris, Erling Svensen, Ståle Pallesen, Siri Waage, Morten Birkeland Nielsen, Erlend Sunde, Bjørn Bjorvatn, Øystein Holmelid, Øystein Vedaa

**Affiliations:** Department of Psychosocial Science, University of Bergen, Bergen, Norway; Department of Health Promotion, Norwegian Institute of Public Health, Bergen, Norway; Department of Psychosocial Science, University of Bergen, Bergen, Norway; Department of Human Resources, Haukeland University Hospital, Bergen, Norway; Department of Psychosocial Science, University of Bergen, Bergen, Norway; Norwegian Competence Center for Sleep Disorders, Haukeland University Hospital, Bergen, Norway; Department of Psychosocial Science, University of Bergen, Bergen, Norway; Norwegian Competence Center for Sleep Disorders, Haukeland University Hospital, Bergen, Norway; Department of Psychosocial Science, University of Bergen, Bergen, Norway; Department of Work Psychology and Physiology, National Institute of Occupational Health, Oslo, Norway; Department of Psychosocial Science, University of Bergen, Bergen, Norway; Norwegian Competence Center for Sleep Disorders, Haukeland University Hospital, Bergen, Norway; Department of Global Public Health and Primary Care, University of Bergen, Bergen, Norway; Department of Psychosocial Science, University of Bergen, Bergen, Norway; Department of Psychosocial Science, University of Bergen, Bergen, Norway; Department of Health Promotion, Norwegian Institute of Public Health, Bergen, Norway

**Keywords:** insomnia, daytime sleepiness, fatigue, shift work, quick returns, healthcare workers, insufficient sleep, daily rest periods

## Abstract

**Study objectives:**

To investigate the effect of a work schedule with abated quick returns (i.e. > 11 hours between two shifts) on insomnia, daytime sleepiness, and work-related fatigue compared to a shift schedule maintaining the usual number of quick returns.

**Methods:**

A two-armed cluster randomized controlled trial including 66 units was conducted at a university hospital in Norway. Units with healthcare workers on rotating shift schedules were randomly assigned to a shift schedule with abated quick returns (intervention) or to continue with a schedule including quick returns as usual (control) for 6 months. Questionnaires assessed symptoms of insomnia (Bergen Insomnia Scale [BIS]), daytime sleepiness (Epworth Sleepiness Scale [ESS]), and work-related fatigue (Revised Swedish Occupational Fatigue Inventory) at baseline and towards the end of the intervention. Data were analyzed using multilevel linear mixed-effects models, and Cohen’s *d* was used to calculate the effect size between groups.

**Results:**

Overall, 1314 healthcare workers (85.2% female) completed the baseline questionnaire (response rate 49.1%), and 552 completed the follow-up questionnaire. The intervention reduced quick returns from an average of 13.2 (SD = 8.7) to 6.7 (SD = 6.0), while the control group’s average remained relatively unchanged from 13.2 (SD = 8.7) to 12.0 (SD = 9.3). Results showed a small improvement in symptoms of insomnia (*BIS; d* = −0.13, *p* = .022) and daytime sleepiness (*ESS*; *d* = −0.14, *p* = .013) in favor of the intervention. No effects were observed on work-related fatigue.

**Conclusions:**

Reducing the number of quick returns in the work schedule resulted in improvements in insomnia and daytime sleepiness. The findings highlight the importance of sufficient daily rest time in the work schedule of healthcare workers.

**Clinical Trial:**

Health Promoting Work Schedules: The Effect of Abolishing Quick Returns (HeWoS); clinicaltrials.gov/ct2/show/NCT04693182; Registered at ClinicalTrials.gov with the identifier NCT04693182.

## Graphical Abstract

**Graphical Abstract:**
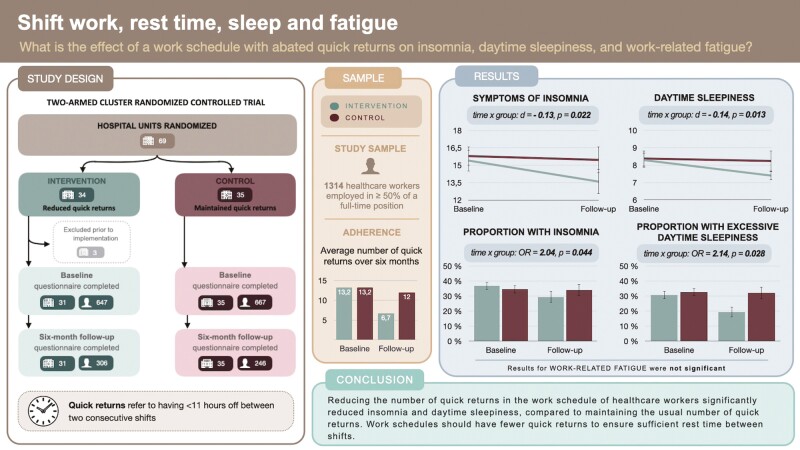

**Statement of Significance**

Although legislations entitle workers to at least 11 hours off between two consecutive shifts, a large proportion of nurses have shift transitions with shorter rest time. In shift work, this is referred to as quick returns. The current study is the first randomized controlled trial to investigate whether abating quick returns impacts self-reported symptoms of insomnia, daytime sleepiness, and work-related fatigue. This study revealed that, compared to the control condition, abating quick returns led to a significant reduction in symptoms of insomnia and less daytime sleepiness among healthcare workers. The findings call attention to the importance of ensuring sufficient time for rest between two consecutive shifts and the benefits of a stricter adherence to working time legislation.

In Western countries, one-fifth of all employees have work hours outside “daytime” hours, commonly referred to as shift work [1–3]. A specific concern with shift work that has received increased attention in recent years, is whether the shift schedule provides employees sufficient time for rest between shifts. According to legislation in many countries, workers are entitled to at least 11 hours off between two consecutive shifts. Despite this, almost one-fourth of European workers report having shift transitions of less than 11 hours each month, which is referred to as quick returns [1, 4]. Quick returns are especially common in the healthcare sector in Scandinavian countries, where between 60 and 80 percent of nurses regularly have less than 11 hours off between two shifts [5, 6].

Most quick returns occur between an evening shift and a day/morning shift the following day, typically limiting the workers’ sleep duration to 5.5 to 6.5 hours, in contrast to 7.0 to 8.0 hours of sleep when they do not have a quick return [4, 7]. Insufficient sleep and misalignment between the endogenous circadian rhythm and the sleep–wake-cycle are assumed to be underlying mechanisms for the adverse health consequences associated with shift work [8, 9]. Furthermore, shifts that cause the greatest disruption of sleep—such as quick returns—appear to be particularly problematic [4, 10–12]. Along with shorter sleep duration, the most acute consequences of quick returns seem to be increased daytime sleepiness and fatigue [4]. A dose-dependent relationship has been identified in studies, where higher frequency of quick returns predicted more adverse outcomes such as poor sleep quality, short sleep duration, difficulties unwinding, exhaustion [13], excessive sleepiness, and fatigue [5, 14]. These sleep-related difficulties and daytime impairments align with symptoms of insomnia. Research indicates that quick returns are linked to a higher likelihood of experiencing insomnia [5]. Furthermore, the challenge of unwinding and the urge to fall asleep swiftly during quick returns could notably influence the development of insomnia, because trying to control or forcibly induce sleep is recognized to worsen and sustain insomnia symptoms [15]. Conversely, two smaller non-randomized intervention studies found that reducing the number of quick returns was associated with improved self-reported sleep and alertness [16], and less tiredness [17].

The abovementioned studies are primarily based on correlational, longitudinal, and survey-based design [5, 13, 14]. In addition, there are few studies on quick returns that combine objective measures of exposure to working time with self-reported outcome variables. The latter is particularly important when studying outcomes that primarily are subjectively experienced, such as symptoms of insomnia, sleepiness, and fatigue. Furthermore, no previous studies have been able to establish a causal relationship between quick returns and these outcomes. A systematic review of interventions to reduce occupational fatigue and sleepiness revealed that none of the included randomized controlled trials addressed quick returns [18]. Furthermore, all previous intervention studies that removed quick returns from the work schedule were non-controlled [16, 17]. In one of the previous intervention studies, the effect might have been confounded by a parallel intervention in which more work time flexibility was introduced [17].

Accordingly, the aim of this cluster randomized controlled trial was to investigate the effect of a work schedule with abated quick returns on symptoms of insomnia, daytime sleepiness, and work-related fatigue, compared to a shift schedule that maintained the usual number of quick returns.

## Materials and Methods

### Study design and procedure

This study presents results from a two-armed cluster randomized controlled trial with a 6-month follow-up. The trial protocol has been published [19] (see Supplementary Table S1 for changes to the protocol). The trial was pre-registered with the Clinical Trials website (NCT04693182) and the Regional Committee for Medical and Health Research Ethics in Western Norway (2020/200386) approved the trial. The reporting of this study adheres to the guidelines outlined in the Consolidated Standards of Reporting Trials (CONSORT) statement for cluster randomized controlled trials [20] and the CONSERVE 2021 statement for trials that were modified due to the COVID-19 pandemic [21].

**Figure 1.**
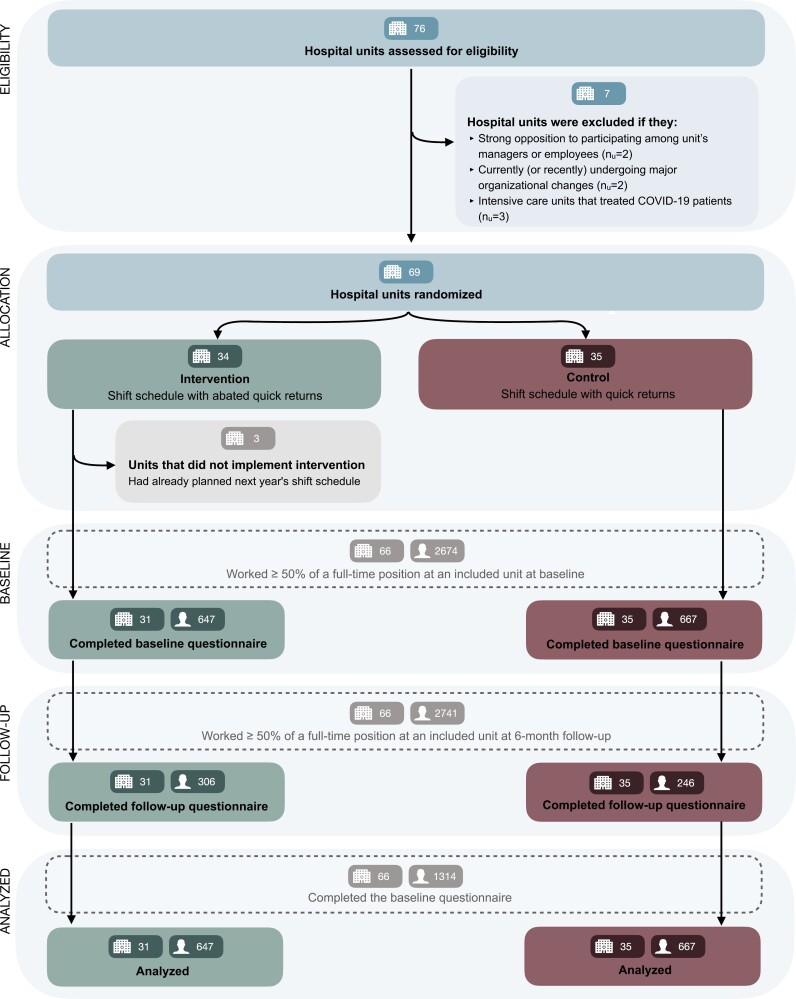
Flow of hospital units and healthcare workers throughout the trial. The invitation to participate was distributed at unit level at both timepoints. Consequently, employees that had quit or changed unit to one that was not included in the trial was no longer available to be invited to the assessment. This made it not possible to identify who were lost to follow-up because they quit or changed to a non-trial unit, and who simply did not want to participate in the follow-up assessment.

Figure 1 presents the flow of participants through the trial. The hospital units, which composed the clusters were randomly assigned to either: (1) a 6-month shift schedule with as few quick returns as possible (intervention), or (2) a 6-month shift schedule that maintained the usual number of quick returns (control). Allocation to the respective groups took place in September 2020. The following autumn, the hospital units planned the 6-month shift schedule in accordance with their assigned condition. The shift schedules were planned 1 year at a time, thereby providing the intervention units with the flexibility to implement the intervention in either the first or the last 6 months of the shift rotation year. To account for potential seasonal effects on the trial outcomes, the control units were matched to the intervention units’ 6-month period based on unit size and subject specialty. The intervention period commenced on January 11th, 2021 for the first units, thereafter, the units had slightly different start times throughout the year, and the last units completed their 6-month intervention period on May 22nd, 2022.

### Study population

The trial was conducted at Haukeland University Hospital, Bergen, Norway. Of the 76 hospital units that had 24-hour staffing, 66 units were deemed eligible for inclusion at the unit level based on the following criteria: (1) the unit had healthcare workers (other than physicians) who worked rotating shifts, (2) the employees had quick returns regularly in their work schedule, and (3) the new 1-year shift schedule was set to commence during the first half of 2021. Physicians were excluded from the study as they have a different shift schedule and compensation scheme than other occupational groups at the hospital. Units were excluded from the study if they: (1) were currently or about to undergo major organizational changes that could affect the trial’s results during the intervention period, or (2) their manager or a significant number of employees strongly objected to participation. Additionally, intensive care units that treated COVID patients during the pandemic were excluded due to the demanding nature of their work and the priority given to preserving life and health, which could make adhering to a new shift schedule challenging (Supplementary Table S1 for changes from the Protocol).

Employees with a contract of at least 50 percent of a full-time position at the included units were invited to complete a digital questionnaire before the first units started their new shift schedule (January 2021), resulting in a baseline measure (invited *N* = 2674). Toward the end of the 6-month intervention period, the employees at the intervention units and their matched control units were invited to complete a follow-up questionnaire (*N* = 2741). As the invitation to participate was distributed at the unit level, employees who no longer worked at the included units were not invited to participate and were therefore unavailable for the research project. The questionnaire was made available to the employees through the hospital’s internal IT system where employees also register their working hours. All included healthcare workers provided informed consent to participate.

### Randomization

The clusters (hospital units) were randomly assigned to one of the two conditions as described above. The required staffing on each shift varied across units, leading to potential baseline differences in the occurrence of quick returns. To address this issue, the units were grouped into 10 strata based on shared characteristics such as emergency functions, mental healthcare functions, maternity care, etc. Each stratum included between 2 and 19 hospital care units. The units were then subjected to stratified randomization in a 1:1 ratio to the two trial conditions. In the strata containing an odd number of units, the remaining units were randomly assigned to one of the conditions. Neither the units nor their employees were masked to the condition they were assigned to, as they actively participated in the planning and implementation of the shift schedule.

After randomization, it was discovered that three units assigned to the intervention group had already planned their shift schedule for the following year. In accordance with the hospital’s human resources department recommendations, these units were excluded from the study as they were not required to re-plan their schedule. A total of 22 healthcare workers changed unit affiliation from baseline to the 6-month follow-up assessment, and 12 of them also changed their study conditions. In the analyses, these healthcare workers were assigned to the unit and randomization group that they were affiliated with at baseline.

#### Intervention group

The intervention aimed to implement a shift schedule without quick returns for a 6-month intervention period. Prior to the trial, healthcare workers at this hospital worked on average three quick returns per month [22]. Although the initial aim of this trial was to eliminate these shifts entirely, due to practical considerations, such as the need to maintain adequate staffing (e.g. due to sickness absence) or unexpected shift swaps, it was not always feasible to achieve complete elimination. Consequently, the intervention aimed to abate the number of quick returns rather than remove them completely. The unit managers in the intervention group received assistance from the hospital’s human resources department in order to plan appropriate schedules with abated quick returns. Examples of schedules without quick returns that aided this work are provided in the trial protocol [19].

#### Control group

The control condition entailed planning shift schedules that maintained the employees’ usual number of quick returns as in the previous year throughout the project period. The term “usual number” of quick returns refers to the common practice at the hospital unit before the intervention, where no explicit changes were made to the work schedule. The units in the control condition were thus not expected to experience any change in the number of quick returns.

### Unwanted negative events or effects

Questions assessing possible unwanted negative events or effects of the intervention were developed for the purpose of this trial and included in the follow-up assessment. Specifically, the questions intended to map if their current work schedule had led to disturbed sleep, more stress, worry, depression, overall less time for recovery between work periods, problems in work–family balance, disrupted social relationships, poorer psychosocial climate at work, experience of reduced quality of care offered to patients, etc. These questions were not intended to serve as “outcome variables,” instead they provided participants a way to give their feedback on the research project. Additionally, the employees received the contact information for the research team through the hospital’s internal website, so that they could report possible unwanted events or effects.

### Measurements

#### Primary outcomes

Symptoms of insomnia were measured using the *Bergen Insomnia Scale* (BIS) [23], which consists of six questions assessing specific symptoms of insomnia. On an 8-point scale between 0 and 7, the healthcare workers stated the number of days they had experienced various sleep problems during the past 3 months. The scores on each item were summed up to a total score ranging from 0 to 42, where higher scores indicate higher levels of insomnia symptoms. Additionally, a dichotomous variable was computed in accordance with the DSM-5 inclusion criteria for insomnia serving as a proxy for insomnia disorder (yes/no) [24]. To score this variable, an item was added asking about the duration of difficulties. The BIS items showed good internal consistency with a Cronbach´s α of .84 at baseline and .85 at follow-up.

Daytime sleepiness was assessed using the *Epworth Sleepiness Scale* (ESS) [25]. Participants were asked to rate the likelihood of dozing off in eight everyday situations on a 4-point Likert scale from “no chance of dozing” (0) to “high chance of dozing” (3). The composite score of all items provided a total score between 0 and 24 where higher scores indicate higher daytime sleepiness. A score of > 10 is considered to reflect excessive daytime sleepiness, and a dichotomous variable (yes/no) was computed. Cronbach´s α was .79 at both baseline and follow-up.

Perceived work-related fatigue was measured using the revised *Swedish Occupational Fatigue Inventory* (rSOFI) [26]. Participants were asked to indicate the degree to which they had experienced 20 different psychological and physical sensations related to fatigue in the past week on a 7-point Likert scale ranging from “not at all” (0) to “a very high degree” (6). The 20 items constitute five dimensions of work-related fatigue: *lack of energy, physical exertion, physical discomfort, lack of motivation,* and *sleepiness.* Each dimension has a total score range of 0 to 6. All the dimensions showed satisfactory to excellent internal consistency at both timepoints (*Lack of energy:* α = .91 and α = .92; *physical exertion:* α = .73 and α = .75; *physical discomfort:* α = .79 and α = .78; *lack of motivation:* α = .86 and α = .88; and *sleepiness:* α = .85 and α = .86 for baseline and follow-up, respectively).

#### Participant characteristics

Baseline demographic information comprising self-reported data on sex (female/male/do not want to answer), years of work experience (indicating number of years), married/cohabitating with partner (yes/no), and presence of children in the household (yes/no) was recorded. Age was calculated based on information about birth year retrieved from the employee register kept by the hospital, with 2021 as reference year. Notably, this information could not be obtained for healthcare workers who were no longer employed at the hospital at the time of data retrieval (July 2022), and these healthcare workers had therefore missing information on age (*n*_intervention_ = 120, *n*_control_ = 103).

#### Adherence

To assess adherence to the intervention of reducing the number of quick returns in the shift schedule, data on working hours was obtained from the local payroll records kept by the hospital. For each participating employee, working hour data were retrieved for the intervention period for the respective unit they worked at when they completed the baseline questionnaire, and for the 6-month period preceding the baseline assessment to serve as a baseline period (July 2020 to January 2021). In line with the definition, *quick returns* were operationalized as transitions between two shifts that permitted <11 hours of rest [4]. The total number of quick returns worked in the 6-month intervention period and the 6-month baseline period, was summarized respectively. Any reduction in the number of quick returns from the baseline period to the intervention period was considered indicative of adherence to the trial protocol. Additionally, for descriptive purposes, the average time between the two shifts in a quick return was calculated for the baseline period and intervention period separately.

### Statistical analysis

#### Sample size

As described in the protocol paper [19], the trial was designed to be able to detect a difference in sickness absence days between the two trial conditions (*n* = 2028). For the sleep and fatigue outcomes investigated in this secondary study, we expected that a smaller sample size would be sufficient to detect potential effects. However, no post hoc power estimations were performed as such estimations could be invalid and misleading [27]. Instead, we interpreted the width and magnitude of the 95% confidence intervals (CI) of the nonsignificant estimates to determine statistical power, in agreement with recommendations [28].

#### Data analysis

Analyses were conducted using IBM SPSS Statistics, V.28.0.1.0 (IBM SPSS Statistics, New York, USA) and STATA/SE V.17.0 (Stata Statistical Software, College Station, TX: StataCorp LLC). The characteristics of the baseline sample and units are presented by randomization group and total sample, whereas characteristics of quick returns are presented by randomization group and time of assessment. Continuous variables are shown as mean and standard deviation (SD), and categorical variables as numbers and percentages. The outcomes investigated in these analyses were symptoms of insomnia (BIS), daytime sleepiness (ESS), and work-related fatigue (rSOFI), of which all were continuous variables. Complementary analyses with dichotomous outcomes of insomnia disorder (yes/no) and excessive daytime sleepiness (yes/no) were also conducted.

To assess the effect of a shift schedule with abated quick returns (intervention) compared to a schedule maintaining the usual number of quick returns (control) on BIS, ESS, and the five rSOFI dimensions, separate multilevel linear mixed-effects models were run, assuming the intention-to-treat principle. In the fixed part of the model, variables for the randomization group, time, and their interaction term were included. The random part of the model included a variable for hospital unit to account for data clustering. Random intercepts were specified for each cluster, allowing for subject-specific deviations from the average trend and cluster-level variability. Missing data were assumed to be missing at random and robust maximum likelihood estimator was employed. The intervention effect was estimated by the group × time interaction-term, which indicates differential change in outcome by group from baseline to follow-up and is reported as coefficient and 95% CI. In line with the trial protocol [19], between-group effect sizes (Cohen’s *d*) were calculated by taking the mean difference in estimated change in scores from baseline to follow-up, divided by the pooled SD at baseline. According to recognized benchmarks, effect sizes (*d*) of 0.8 are regarded as large, 0.5 as moderate, and 0.2 as small, respectively [29]. Significance level was set to 95 percent.

To test the robustness of the results, sensitivity analyses were conducted. As some data at follow-up may have been missing not at random, analyses where missing scores at follow-up were replaced by baseline values for each respective individual were conducted (i.e. last observation carried forward). The results of these analyses are presented in Supplementary Table S2. In addition, analyses where the healthcare workers who changed unit affiliation from baseline to follow-up (*n* = 22) were omitted were also run. These results are shown in Supplementary Table S3.

Complementary analyses using the dichotomous scoring of BIS and ESS as outcomes were conducted to assess the effect of the intervention on a proxy for insomnia disorder and excessive daytime sleepiness. For this purpose, separate mixed-effects logistic regression models were run in the intention-to-treat sample with time, group, and their interaction in the fixed part of the model, and hospital unit in the random part to account for data clustering. The group × time-interaction estimated the intervention effect and is presented as odds ratio (OR) with 95% CI.

## Results

The flow of participants in the trial is illustrated in Figure 1. Of the 69 hospital units included, 34 were allocated to the intervention group. Of those, three units were excluded prior to implementation. The remaining 35 hospital units made up the control group. Thus, 66 units participated in the trial. In total, 1314 healthcare workers completed the questionnaire at baseline (response rate 49.1%), and of those, 552 completed the questionnaire at 6-month follow-up. Notably, the number of participants at follow-up was influenced by the fact that some employees had quit or changed the unit to one that was not included in the trial and were thus no longer available to be invited to the assessment. It was not possible to identify who quit or changed to a non-trial unit, and who simply did not want to participate in the follow-up assessment.

### Sample characteristics

Table 1 shows descriptive characteristics of the study sample at baseline. There were some differences in the sex distribution between the two groups, with the control group consisting of more males than the intervention group (17.2% and 9.9%, respectively). On average, the sample had 12.2 years of shift work experience. Furthermore, two-thirds of the workers were married or cohabitating with a partner and over one-third had children living in their household.

**Table 1.** Baseline Characteristics of Hospital Units and Healthcare Workers (*n* = 1314)

	Shift schedule with abated quick returns (units = 31, *n* = 647) (intervention)	Shift schedule with quick returns as usual (units = 35, *n* = 667) (control)	Total (units = 66, *n* = 1314)
**U** **ni** **ts**			
Cluster size	20.9 (9.7)	19.1 (8.2)	19.9 (8.9)
**Healthcare** **workers**			
Age^*^, years	38.3 (12.8)	36.6 (11.7)	37.5 (12.3)
Years of experience	13.0 (10.9)	11.5 (9.7)	12.2 (10.4)
Sex			
Female	577 (89.2%)	543 (81.4%)	1120 (85.2%)
Male	64 (9.9%)	115 (17.2%)	179 (13.6%)
Do not want to answer	6 (0.9%)	9 (1.4%)	15 (1.1%)
Married or cohabiting with partner			
Yes	435 (67.2%)	432 (64.8%)	867 (66.0%)
No	212 (32.8%)	235 (35.2%)	447 (34.0%)
Children living in household			
Yes	247 (38.2%)	234 (35.1%)	481 (36.6%)
No	400 (61.8%)	433 (64.9%)	833 (63.4%)

Data are presented as mean (SD) or *n* (%).

^*^Due to missing data from the hospital’s employee information register, n was somewhat lower for the variable on age, N_age_ = 1091.

### Summary of intervention delivery

As shown in Table 2, during the baseline period, the healthcare workers in both conditions worked on average the same number of quick returns (mean = 13.2, SD = 8.7 for both groups). However, the intervention group halved the average number of quick returns worked down to an average of 6.7 (SD = 6.0), while the control group maintained a relatively similar number of quick returns with an average of 12.0 (SD = 9.3) during the intervention period. Of the healthcare workers in the intervention group, 71.8% reduced the number of quick returns from the baseline period to the follow-up period. In instances of quick returns, the average time of hours between the shifts remained consistent at approximately 9 hours irrespective of the study group or time of assessment.

**Table 2.** Number of Quick Returns and Time Between Shifts in a Quick Return During the Baseline Period and Intervention Period

	Shift schedule with abated quick returns (intervention)		Shift schedule with quick returns as usual (control)	
	Baseline period^b^ (*n* = 638)	Intervention period^b^ (*n* = 630)	Baseline period^b^ (*n* = 666)	Intervention period^b^ (*n* = 637)
Number of quick returns^a^	13.2 (8.7)	6.7 (6.0)	13.2 (8.7)	12.0 (9.3)
Time between shifts in a quick return^a^ in hours and minutes	09:10 (00:22)	09:07 (00:28)	09:06 (00:35)	09:07 (00:28)

Data are presented as mean (SD).

^a^Quick returns refer to < 11 hours of rest between two consecutive shifts.

^b^The number of participants deviates from the number who answered the questionnaire as shown in Table 1, since the present table show register data obtained from the hospital on all workers who answered the baseline assessment.

### Effects on symptoms of insomnia, daytime sleepiness, and work-related fatigue

Table 3 presents the results of the intention-to-treat multilevel linear mixed-effects model analyses on symptoms of insomnia, daytime sleepiness, and work-related fatigue. The results showed that the intervention group had a significantly larger reduction in symptoms of insomnia, compared to the control group (Cohen’s *d* = −0.13, *p* = .022). Similar results were found for daytime sleepiness, where the results showed a significantly larger reduction in daytime sleepiness in favor of the intervention group (Cohen’s *d* = −0.14, *p* = .013). Regarding the work-related fatigue outcomes, the results did not reveal any significant interaction effects for any of the five dimensions.

**Table 3.** Results From the Intention-to-Treat Analysis on symptoms of insomnia, daytime sleepiness and work-related fatigue (*n* = 1314)

	Shift schedule with abated quick returns (intervention)		Shift schedule with quick returns as usual (control)		Intervention effect		
	No.	Mean* (SE)	No.	Mean* (SE)	Coefficient (95% CI)	Cohen’s d	*P*-value
Bergen Insomnia Scale							
Baseline	647	15.40 (0.46)	667	15.79 (0.41)	**1.48** (0.22 to 2.75)	**−0.13**	**.022**
Follow-up	306	13.59 (0.54)	246	15.46 (0.58)			
Epworth Sleepiness Scale							
Baseline	647	8.29 (0.21)	667	8.38 (0.23)	**0.75** (0.16 to 1.35)	**−0.14**	**.013**
Follow-up	306	7.40 (0.25)	246	8.24 (0.30)			
*Revised Swedish Occupational Fatigue Inventory*							
Lack of energy							
Baseline	647	1.86 (0.10)	667	1.98 (0.11)	0.03 (−0.23 to 0.30)	−0.01	.798
Follow-up	306	1.99 (0.13)	246	2.15 (0.13)			
Physical exertion							
Baseline	647	0.81 (0.05)	667	0.85 (0.04)	0.13 (−0.05 to 0.31)	−0.11	.144
Follow-up	306	0.90 (0.06)	246	1.07 (0.90)			
Physical discomfort							
Baseline	647	1.47 (0.07)	667	1.48 (0.06)	0.14 (−0.06 to 0.34)	−0.09	.164
Follow-up	306	1.43 (0.08)	246	1.59 (0.09)			
Lack of motivation							
Baseline	647	1.13 (0.07)	667	1.22 (0.06)	0.04 (−0.17 to 0.26)	−0.02	.685
Follow-up	306	1.18 (0.10)	246	1.31 (0.08)			
Sleepiness							
Baseline	647	1.89 (0.08)	667	1.88 (0.07)	0.13 (−0.08 to 0.33)	−0.07	.239
Follow-up	306	1.72 (0.09)	246	1.84 (0.09)			

Mean, estimated mean; SE, standard error; CI, confidence interval. Values in bold indicate statistically significant results, with p < .005.

Additionally, sensitivity analyses with last observation carried forward for missing values at follow-up were carried out, as well as analyses where the healthcare workers who changed unit affiliation between timepoints were omitted. These analyses displayed similar results as in the intention-to-treat analyses with a larger reduction in symptoms of insomnia and daytime sleepiness in the intervention group compared to the control group. Notably, the between-group effect sizes were the same or lower in the sensitivity analysis compared to the intention-to-treat analysis (Supplementary Table S2 and S3).

In Table 4, the results from the intention-to-treat mixed-effects logistic regression model analyses on insomnia disorder and excessive daytime sleepiness are reported. The results show that the healthcare workers in the control group had a significantly higher odds of insomnia (OR = 2.04, *p* = .044) and excessive daytime sleepiness (OR = 2.14, *p* = .028) compared to the intervention group. Moreover, descriptive statistics show that at baseline, 36.8% and 34.5% of the healthcare workers met the DSM-5 inclusion criteria for insomnia in the intervention and control group, respectively. However, at follow-up, 29.4% of the healthcare workers in the intervention group had insomnia disorder, whereas the proportion in the control group remained unchanged at around 34%. Similarly, the proportion of healthcare workers with excessive daytime sleepiness decreased from 30.6% to 19.3% in the intervention group from baseline to follow-up, while the corresponding proportion in the control group remained around 32% at both timepoints.

**Table 4.** Results from the intention-to-treat analysis on insomnia disorder and excessive daytime sleepiness (*n* = 1314)

	Shift schedule with abated quick returns (intervention)		Shift schedule with quick returns as usual (control)		Intervention effect	
	Baseline (*n* = 647)	Follow-up (*n* = 306)	Baseline (*n* = 667)	Follow-up (*n* = 246)	OR (95% CI)	*P*-value
Insomnia disorder ^a^						
Yes	36.8%	29.4%	34.5%	34.1%	**2.04** (0.62 to 3.45)	**.044**
No	63.2%	70.6%	65.5%	65.9%		
Excessive daytime sleepiness ^b^						
Yes	30.6%	19.3%	32.7%	32.1%	**2.14** (0.69 to 3.60)	**.028**
No	69.4%	80.7%	67.3%	67.9%		

Data are presented as observed proportion with insomnia (yes/no) and excessive daytime sleepiness (yes/no) across time and randomization group.

OR, odds ratio; CI, confidence interval. Values in bold indicate statistically significant results, with p < .005.

^a^A proxy for insomnia disorder measured using the Bergen Insomnia Scale (BIS). Healthcare workers had to have nocturnal symptoms (difficulties with sleep initiation, sleep maintenance, early morning awakening, and nonrestorative sleep) at least three nights a week for at least 3 months, and daytime symptoms (daytime impairment and dissatisfaction with sleep) at least 3 days a week for at least 3 months to meet the DSM-5 criteria for insomnia.

^b^Measured using Epworth Sleepiness Scale (ESS) where a total score of > 10 indicate the cutoff for excessive daytime sleepiness.

### Unwanted negative events or effects

During the project period, no healthcare workers contacted the research team to report unwanted negative events or effects. Furthermore, Supplementary Table S4 presents an overview of the possible negative events or effects reported by the healthcare workers in the follow-up assessment. In general, most healthcare workers in both groups stated that they did not experience any of the outlined negative events or effects. However, slightly more healthcare workers in the intervention group reported their new shift schedule as more unfavorable and less flexible in terms of being able to swap shifts, and experienced greater difficulties planning family and leisure activities, compared to the control group. In addition, somewhat more workers in the intervention group also experienced that the continuity of care for the patients became worse, compared to the control group.

## Discussion

The purpose of this cluster RCT was to investigate the effect of a work schedule with abated quick returns on symptoms of insomnia, daytime sleepiness, and work-related fatigue, compared to a shift schedule that maintained the usual number of quick returns. Overall, the results showed that halving the number of quick returns in the work schedule led to a larger reduction in self-reported symptoms of insomnia and daytime sleepiness compared to the control group. However, the intervention had no effect on self-reported work-related fatigue. All effect sizes were small, yet remained significant in the sensitivity analyses.

Previous studies have demonstrated that quick returns are associated with impaired sleep and excessive sleepiness [4, 5, 13, 16, 17]. Our results significantly add to the existing evidence by being based on the first RCT to show that insomnia and daytime sleepiness are improved when quick returns are reduced in the work schedule. The effect-size improvements on insomnia symptoms and daytime sleepiness were small, which may question the practical implications of the findings. However, it should be noted that the current intervention only intended to make minor changes to the working hour arrangements for an initially healthy group of people. Moreover, the intervention is not a clinical treatment for people who have preidentified sleep difficulties. In this sense, achieving such a clear and consistent effect on the sleep and functioning of the employees is quite noteworthy. Indeed, according to the prevention paradox, small risk reductions for many people may have a higher public health impact and economic gains than large risk reductions for those most at risk [30]. In addition, the minor work schedule changes implemented in this trial can be considered free of financial costs and a readily available measures that positively affect shift workers sleep and functioning.

The literature thus far has indicated a dose–response relationship between quick returns and sleep and fatigue, where a high number of quick returns is associated with increased sleep disturbances and fatigue [5, 13, 14]. Although this trial was not designed to differentiate between different levels of exposure, the intervention was successful only in halving the number of quick returns in the intervention group. It remains unclear what the effect would have been if quick returns were completely abolished. As indicated by others, a complete abolition of quick returns may not be ideal for all outcomes [31, 32], as many may find that quick returns benefit other aspects of work (e.g. longer consecutive periods off work) and leisure beyond sleep and daytime functioning. It remains to be investigated whether there is a linear relationship between the number of quick returns and sleep difficulties/sleepiness, or whether there may also be advantages to maintain a certain number of quick returns. In addition, there also might be individual characteristics (e.g. sleep need and sleep flexibility) that moderate the effects of abating quick returns, which should be elucidated.

It was somewhat surprising that no effects of the intervention were found on work-related fatigue. Previous studies have indicated a proportional relationship between number of quick returns and the level of fatigue [5, 14]. Accordingly, it was hypothesized that a reduction in quick returns would have corresponding favorable effects on work-related fatigue. However, in this trial, an instrument that focuses on the momentary and bodily features of fatigue [26] was utilized, while previous studies have used an instrument that focuses on long-term features of fatigue and its impact on functioning [5, 14]. Hence, it can be speculated that the instrument used in this trial was not successful in detecting relevant features of fatigue related to quick returns. This may be reflected in the low estimated mean scores and narrow 95% CI of the rSOFI dimensions across both groups and timepoints, indicating that the healthcare workers in this sample generally experienced a low degree of fatigue. This contrasts with a previous study that found a high prevalence (35.5 to 43.4 percent) of excessive fatigue among Norwegian nurses [5]. Additionally, the confidence intervals of the coefficients from the rSOFI models were narrow (ranging from −0.23 to 0.33), indicating that any potential effect measured by this instrument was likely too small to be detected in our sample [28]. This should be considered when interpreting the results from this trial.

### Strengths and limitations

The major strengths of this trial are its randomized controlled design and the relatively large sample size, which enabled determination of the causal relationship between reduction of quick returns and insomnia, sleepiness, and fatigue as outcomes. The large sample size also provides sufficient statistical power to draw unambiguous conclusions. Additionally, a notable strength is the utilization of register-based data to determine adherence to the intervention.

However, there are also some limitations to the trial that should be mentioned. The use of self-reported measures may be susceptible to biases related to measurement and individual subjectivity. Nevertheless, the instruments used are validated and have demonstrated satisfactory psychometric properties in previous studies. It should also be noted that the measured outcomes are primarily subjective in nature and should therefore be measured using self-reported instruments. While the study’s randomized design aimed to evenly distribute comorbid sleep disorders across groups, the exclusion of explicit evaluation of these conditions, such as sleep apnea and parasomnias, represents a limitation that may affect the interpretation of the findings. Furthermore, the trial was conducted in a naturalistic setting. Therefore, a strict experimental manipulation which is typically achieved in lab studies was not feasible. The intervention group failed to completely remove quick returns from the work schedule, achieving only a halving of their occurrence. Thus, an objection that can be raised is that the intervention was incomplete. However, there are studies that indicate a threshold regarding the effect of quick returns, where having many quick returns is negative for health, but having a few might be advantageous [31, 32]. The results of the present study indicated that halving the number of quick returns had significant beneficial effects. It is conceivable that the reduction achieved in the number of quick returns approached the aforementioned threshold, as indicated in previous studies. Further, the low response rate (49%) at baseline is a limitation. It is unclear what this means for the generalizability of the findings. However, obtaining answers from about half of those asked should probably be considered acceptable as population-based surveys often do not obtain response rates higher than 40 percent [33]. There are also studies that show that increasing the response rate does not necessarily change the response pattern and the conclusion from the surveys [34]. There was also a large dropout from baseline to follow-up, which contributes to the uncertainty in terms of the generalizability of the findings despite consistent findings in intention-to-treat analyses and sensitivity analyses. To evaluate potential baseline outcome differences between healthcare workers based on follow-up questionnaire completion, cluster-adjusted linear regression analyses for each continuous outcome, stratifying participants by their response status (completed at baseline only vs. completed at both baseline and follow-up) were performed. The analysis showed no significant differences between the groups, indicating that attrition is unlikely to introduce significant bias into the results (refer to Supplementary Table S5).

Another limitation of the present study was that the sample mainly consisted of female healthcare workers, which may limit the generalizability of the results to other industries and both sexes. Yet, the sample is representative of healthcare workers in general, who mainly consist of females [22]. It is worth noting that the sex distribution in the two randomization groups was somewhat different. The general recommendation in RCT studies is to not adjust for baseline differences that were not hypothesized to act as moderators in a prepublished registration/protocol [20]. Accordingly, the results are primarily reported without adjusting for sex. However, the supplementary material includes a table showing the results adjusted for sex, of which there were no differences compared to the main analysis (Supplementary Table S4).

### Conclusion and implications

Reducing the number of quick returns in the work schedule of healthcare workers led to a larger reduction of symptoms of insomnia and daytime sleepiness, compared to the control group that continued with the usual number of quick returns. These findings highlight the importance of ensuring adequate time for rest between two consecutive shifts, in line with legislation in many countries. As such, policymakers and employers should take action to reduce the extensive use of quick returns, particularly in the healthcare sector. Notably, the intervention implemented in the current study only involved halving the number of quick returns, yet still produced a significant improvement in the participants’ sleep and functioning. Further research is needed to establish whether a further reduction or complete abolishment of quick returns is advantageous, and if any of the potential benefits are contingent upon individual characteristics.

## Supplementary Material

zsae086_suppl_Supplementary_Materials

## Data Availability

The ethical approval of this trial does not allow for sharing of the individual-level data that support the findings. However, synthetic data can be made available from the corresponding author upon reasonable request.
