# Supplementary material for: Effects of a work schedule with abated quick returns on insomnia, sleepiness, and work-related fatigue: results from a large-scale cluster randomized controlled trial
Source: Sleep. 2024 Apr 6;47(7):zsae086. doi: 10.1093/sleep/zsae086 (PMC11236942; doi:10.1093/sleep/zsae086)
Supplement: zsae086_suppl_Supplementary_Materials [file zsae086_suppl_supplementary_materials.docx]

**Effects of a work schedule with abated quick returns on insomnia, sleepiness and work-related fatigue: Results from a large-scale cluster randomized controlled trial**

Ingebjørg Louise Rockwell Djupedal^1,2^, Anette Harris^1^, Erling Svensen^3^, Ståle Pallesen^1,4^, Siri Waage^1,4^, Morten Birkeland Nielsen^1,5^, Erlend Sunde^1^, Bjørn Bjorvatn^4,6^ Øystein Holmelid^1^, Øystein Vedaa^1,2^

^1^ Department of Psychosocial Science, University of Bergen, Bergen, Norway
^2^ Department of Health Promotion, Norwegian Institute of Public Health, Norway
^3^ Department of Human Resources, Haukeland University Hospital, Bergen

^4^ Norwegian Competence Center for Sleep Disorders, Haukeland University Hospital, Bergen, Norway
^5^ Department of Work Psychology and Physiology, National Institute of Occupational Health, Oslo, Norway

^6^ Department of Global Public Health and Primary Care, University of Bergen, Bergen, Norway

**Correspondence to**: Ingebjørg Louise Rockwell Djupedal, Department of Psychosocial Science, University of Bergen, Bergen, Norway; e-mail: [Ingebjorg.Djupedal@uib.no](mailto:Ingebjorg.Djupedal@uib.no)

**SUPPLEMENTARY TABLES**

| **Table S1.** Table outlining the changes to the study during the project period due to the COVID-19 pandemic. | | |
| --- | --- | --- |
| **Change to the study** | **Details of the change** | **Date of change** |
| Change in study  population | The intensive care units at Haukeland University hospital were responsible for the treatment of patients with covid-infection during the pandemic. Prior to the randomization, these units provided feedback that it would be difficult to implement and comply to a new shift schedule as the units were in a pressured work situation where considerations of life and health were prioritized. The intensive care units were therefore excluded from the study. | August 2020 |

| **Table S2.** Results from the sensitivity analyses on primary outcomes where missing at follow-up were replaced by baseline values (i.e., last observation carried forward) (n=1314). | | | | | | | | | |
| --- | --- | --- | --- | --- | --- | --- | --- | --- | --- |
|  | **Shift schedule with abated quick returns** (intervention) | |  | **Shift schedule with quick returns**  (control) | |  | **Intervention effect** | | |
|  | No. | Mean* (SE) |  | No. | Mean* (SE) |  | Coefficient  (95% CI) | Cohen’s d | P-value |
| **Bergen Insomnia Scale** | | | | | | | | | |
| Baseline | 647 | 15.40 (0.46) |  | 667 | 15.79 (0.41) |  | **0.80**  (0.26 to 1.34) | **-0.07** | **0.004** |
| Follow-up | 306 | 14.51 (0.45) |  | 246 | 15.69 (0.42) |  |  |  |  |
| **Epworth Sleepiness Scale** | | | | | | | | | |
| Baseline | 647 | 8.30 (0.21) |  | 667 | 8.38 (0.23) |  | **0.31**  (0.06 to 0.58) | **-0.05** | **0.016** |
| Follow-up | 306 | 7.89 (0.21) |  | 246 | 8.29 (0.23) |  |  |  |  |
| **Revised Swedish Occupational Fatigue Inventory** | | | | | | | | |  |
| *Lack of energy* |  |  |  |  |  |  |  |  |  |
| Baseline | 647 | 1.86 (0.10) |  | 667 | 1.99 (0.11) |  | -0.01  (-0.13 to 0.10) | -0.01 | 0.810 |
| Follow-up | 306 | 1.93 (0.11) |  | 246 | 2.05 (0.10) |  |  |  |  |
| *Physical exertion* |  |  |  |  |  |  |  |  |  |
| Baseline | 647 | 0.81 (0.05) |  | 667 | 0.85 (0.04) |  | 0.03  (-0.05 to 0.10) | -0.02 | 0.464 |
| Follow-up | 306 | 0.86 (0.05) |  | 246 | 0.92 (0.05) |  |  |  |  |
| *Physical discomfort* |  |  |  |  |  |  |  |  |  |
| Baseline | 647 | 1.47 (0.07) |  | 667 | 1.48 (0.06) |  | 0.04  (-0.05 to 0.13) | -0.02 | 0.378 |
| Follow-up | 306 | 1.46 (0.07) |  | 246 | 1.51 (0.07) |  |  |  |  |
| *Lack of motivation* |  |  |  |  |  |  |  |  |  |
| Baseline | 647 | 1.13 (0.07) |  | 667 | 1.22 (0.06) |  | -0.00  (-0.10 to 0.09) | 0 | 0.946 |
| Follow-up | 306 | 1.16 (0.07) |  | 246 | 1.25 (0.06) |  |  |  |  |
| *Sleepiness* |  |  |  |  |  |  |  |  |  |
| Baseline | 647 | 1.89 (0.08) |  | 667 | 1.88 (0.07) |  | 0.04  (-0.05 to 0.13) | -0.01 | 0.430 |
| Follow-up | 306 | 1.83 (0.08) |  | 246 | 1.85 (0.07) |  |  |  |  |
| **Mean: Estimated mean, SE: Standard error, CI: confidence interval*  Values in **bold** indicate statistically significant results, with p < .005. | | | | | | | | | |

| **Table S3.** Results from intention-to-treat analysis on primary outcomes where participants who changed unit affiliation from baseline to follow-up (n=1292) were omitted. | | | | | | | | | | |
| --- | --- | --- | --- | --- | --- | --- | --- | --- | --- | --- |
|  | **Shift schedule with abated quick returns** (intervention) | |  | **Shift schedule with quick returns**  (control) | |  |  | **Intervention effect** | | |
|  | No. | Mean* (SE) |  | No. | Mean* (SE) |  |  | Coefficient  (95% CI) | Cohen’s d | P-value |
| **Bergen Insomnia Scale** | | | | | | | | | | |
| Baseline | 635 | 15.35 (0.47) |  | 657 | 15.80 (0.41) |  |  | **1.53**  (0.26 to 2.80) | **-0.14** | **0.019** |
| Follow-up | 294 | 13.57 (0.57) |  | 236 | 15.55 (0.59) |  |  |  |  |  |
| **Epworth Sleepiness Scale** | | | | | | | | | | |
| Baseline | 635 | 8.30 (0.22) |  | 657 | 8.39 (0.23) |  |  | **0.72**  (0.11 to 1.32) | **-0.12** | **0.020** |
| Follow-up | 294 | 7.44 (0.25) |  | 236 | 8. 24 (0.32) |  |  |  |  |  |
| **Revised Swedish Occupational Fatigue Inventory** | | | | | | | | |  | |
| *Lack of energy* |  |  |  |  |  |  |  |  |  |  |
| Baseline | 635 | 1.87 (0.10) |  | 657 | 1.98 (0.11) |  |  | 0.09  (-0.16 to 0.34) | -0.04 | 0.487 |
| Follow-up | 294 | 1.96 (0.13) |  | 236 | 2.16 (0.13) |  |  |  |  |  |
| *Physical exertion* |  |  |  |  |  |  |  |  |  |  |
| Baseline | 635 | 0.82 (0.05) |  | 657 | 0.85 (0.04) |  |  | 0.13  (-0.05 to 0.31) | -0.12 | 0.146 |
| Follow-up | 294 | 0.90 (0.06) |  | 236 | 1.07 (0.09) |  |  |  |  |  |
| *Physical discomfort* |  |  |  |  |  |  |  |  |  |  |
| Baseline | 635 | 1.47 (0.07) |  | 657 | 1.48 (0.06) |  |  | 0.15  (-0.05 to 0.34) | -0.08 | 0.136 |
| Follow-up | 294 | 1.42 (0.08) |  | 236 | 1.57 (0.09) |  |  |  |  |  |
| *Lack of motivation* |  |  |  |  |  |  |  |  |  |  |
| Baseline | 635 | 1.14 (0.07) |  | 657 | 1.22 (0.06) |  |  | 0.07  (-0.14 to 0.28) | -0.03 | 0.534 |
| Follow-up | 294 | 1.17 (0.10) |  | 236 | 1.31 (0.08) |  |  |  |  |  |
| *Sleepiness* |  |  |  |  |  |  |  |  |  |  |
| Baseline | 635 | 1.89 (0.08) |  | 657 | 1.87 (0.07) |  |  | 0.14  (-0.06 to 0.35) | -0.07 | 0.174 |
| Follow-up | 294 | 1.71 (0.09) |  | 236 | 1.83 (0.09) |  |  |  |  |  |
| ** Mean: Estimated margin mean, SE: Standard error, CI: confidence interval*  Values in **bold** indicate statistically significant results, with p < .005. | | | | | | | | | | |

| **Table S4.** Frequency distribution and total number of responses to questions about possible unwanted negative events or effects due to the shift schedule the last few months. Responses were given at the follow-up assessment (n=552). | | |
| --- | --- | --- |
|  | **Shift schedule with abated quick returns**  (units=31, n=306) | **Shift schedule with quick returns**  (units=35, n=246) |
| I had more trouble sleeping | | |
| *Not at all* | 225 (73.5) | 149 (60.6) |
| *Little* | 37 (12.1) | 38 (15.4) |
| *Somewhat* | 23 (7.5) | 29 (11.8) |
| *To some extent* | 11 (3.6) | 19 (7.7) |
| *To a great extent* | 10 (3.3) | 11 (4.5) |
| I became more stressed | | |
| *Not at all* | 224 (73.2) | 169 (68.7) |
| *Little* | 43 (14.1) | 32 (13.0) |
| *Somewhat* | 19 (6.2) | 25 (10.2) |
| *To some extent* | 15 (4.9) | 16 (6.5) |
| *To a great extent* | 5 (1.6) | 4 (1.6) |
| I became more anxious/restless | | |
| *Not at all* | 270 (88.2) | 192 (78.0) |
| *Little* | 14 (4.6) | 20 (8.1) |
| *Somewhat* | 9 (2.9) | 24 (9.8) |
| *To some extent* | 11 (3.6) | 8 (3.3) |
| *To a great extent* | 2 (0.7) | 2 (0.8) |
| I became more depressed/sad | | |
| *Not at all* | 269 (87.9) | 186 (75.6) |
| *Little* | 13 (4.2) | 29 (11.8) |
| *Somewhat* | 13 (4.2) | 19 ((7.7) |
| *To some extent* | 9 (2.9) | 10 (4.1) |
| *To a great extent* | 2 (0.7) | 2 (0.8) |
| I experienced greater hopelessness | | |
| *Not at all* | 259 (84.6) | 196 (79.7) |
| *Little* | 20 (6.5) | 26 (10.6) |
| *Somewhat* | 14 (4.6) | 16 (6.5) |
| *To some extent* | 10 (3.3) | 6 (2.4) |
| *To a great extent* | 3 (1.0) | 2 (0.8) |
| I experienced more unpleasant feelings | | |
| *Not at all* | 265 (86.6) | 187 (76.0) |
| *Little* | 21 (6.9) | 29 (11.8) |
| *Somewhat* | 5 (1.6) | 20 (8.1) |
| *To some extent* | 11 (3.6) | 7 (2.8) |
| *To a great extent* | 4 (1.3) | 3 (1.2) |
| I experienced that my general condition worsened | | |
| *Not at all* | 270 (88.2) | 197 (80.1) |
| *Little* | 15 (4.9) | 17 (6.9) |
| *Somewhat* | 9 (2.9) | 22 (8.9) |
| *To some extent* | 10 (3.3) | 7 (2.8) |
| *To a great extent* | 2 (0.7) | 3 (1.2) |
| I experienced less rest time between my work periods | | |
| *Not at all* | 244 (79.7) | 166 (67.5) |
| *Little* | 20 (6.5) | 30 (12.2) |
| *Somewhat* | 23 (7.5) | 18 (7.2) |
| *To some extent* | 9 (2.9) | 19 (7.7) |
| *To a great extent* | 10 (3.3) | 13 (5.3) |
| I experienced a poorer balance between work and leisure | | |
| *Not at all* | 178 (58.2) | 161 (65.4) |
| *Little* | 50 (16.3) | 30 (12.2) |
| *Somewhat* | 31 (10.1) | 26 (10.6) |
| *To some extent* | 25 (8.2) | 20 (8.1) |
| *To a great extent* | 22 (7.2) | 9 (3.7) |
| I experienced my shift schedule as more unfavorable | | |
| *Not at all* | 147 (48.0) | 158 (64.2) |
| *Little* | 61 (19.9) | 39 (15.9) |
| *Somewhat* | 33 (10.8) | 27 (11.0) |
| *To some extent* | 34 (11.1) | 10 (4.1) |
| *To a great extent* | 31 (10.1) | 12 (4.9) |
| I experienced less flexibility in terms of being able to swap shifts | | |
| *Not at all* | 156 (51.0) | 173 (70.3) |
| *Little* | 54 (17.6) | 34 (13.8) |
| *Somewhat* | 31 (10.1) | 22 (8.9) |
| *To some extent* | 24 (7.8) | 8 (3.3) |
| *To a great extent* | 41 (13.4) | 9 (3.7) |
| I had greater relational difficulties | | |
| *Not at all* | 267 (87.3) | 203 (82.5) |
| *Little* | 16 (5.2) | 21 (8.5) |
| *Somewhat* | 11 (3.6) | 15 (6.1) |
| *To some extent* | 9 (2.9) | 5 (2.0) |
| *To a great extent* | 3 (1.0) | 2 (0.8) |
| I experienced a worse psychosocial climate at work | | |
| *Not at all* | 237 (77.5) | 194 (78.9) |
| *Little* | 38 (12.4) | 24 (9.8) |
| *Somewhat* | 13 (4.2) | 18 (7.3) |
| *To some extent* | 11 (3.6) | 7 (2.8) |
| *To a great extent* | 7 (2.3) | 3 (1.2) |
| I experienced that the quality of care for the patients deteriorated | | |
| *Not at all* | 225 (73.5) | 194 (78.9) |
| *Little* | 37 (12.1) | 24 (9.8) |
| *Somewhat* | 20 (6.5) | 17 (6.9) |
| *To some extent* | 15 (4.9) | 9 (3.7) |
| *To a great extent* | 9 (2.9) | 2 (0.8) |
| I experienced that the continuity of care for the patients became worse | | |
| *Not at all* | 175 (57.2) | 188 (76.4) |
| *Little* | 58 (19.0) | 21 (8.5) |
| *Somewhat* | 33 (10.8) | 20 (8.1) |
| *To some extent* | 23 (7.5) | 14 (5.7) |
| *To a great extent* | 17 (5.6) | 3 (1.2) |
| I found that I made more mistakes at work | | |
| *Not at all* | 279 (91.2) | 211 (85.8) |
| *Little* | 14 (4.6) | 21 (8.5) |
| *Somewhat* | 9 (2.9) | 12 (4.9) |
| *To some extent* | 4 (1.9) | 2 (0.8) |
| *To a great extent* | 0 (0.0) | 0 (0.0) |
| I was less able to rest and recover when I had time off | | |
| *Not at all* | 228 (74.5) | 176 (71.5) |
| *Little* | 29 (9.5) | 28 (11.4) |
| *Somewhat* | 22 (7.2) | 20 (8.1) |
| *To some extent* | 16 (5.2) | 15 (6.1) |
| *To a great extent* | 11 (3.6) | 7 (2.8) |
| I experienced greater difficulties in planning my family and leisure activities | | |
| *Not at all* | 192 (62.7) | 174 (70.7) |
| *Little* | 45 (14.7) | 31 (12.6) |
| *Somewhat* | 21 (6.9) | 21 (8.5) |
| *To some extent* | 27 (8.8) | 10 (4.1) |
| *To a great extent* | 21 (6.9) | 10 (4.1) |
| *Data are presented as n (%).* | | |

| **Table S5.** Results from the cluster-adjusted linear regression analyses on primary outcomes at baseline, stratified by response status (completed questionnaire at baseline only vs. completed questionnaires at both baseline and follow-up) (n=1314). | | | | | | | | |
| --- | --- | --- | --- | --- | --- | --- | --- | --- |
|  | **Completed at baseline only** | |  | **Completed at both baseline and follow-up** | |  | **Cluster-adjusted linear regression** | |
|  | No. | Mean* (SE) |  | No. | Mean* (SE) |  | Coefficient  (95% CI) | P-value |
| **Bergen Insomnia Scale** | 762 | 15.56 (0.36) |  | 552 | 15.54 (0.41) |  | -0.01 (-0.92 to 0.89) | 0.980 |
| **Epworth Sleepiness Scale** | 762 | 8.35 (0.17) |  | 552 | 8.42 (0.22) |  | 0.07  (-0.41 to 0.56) | 0.763 |
| **Revised Swedish Occupational Fatigue Inventory** |  |  |  |  |  |  |  |  |
| *Lack of energy* | 762 | 1.18 (0.06) |  | 552 | 1.14 (0.06) |  | -0.04  (-0.18 to 0.09) | 0.515 |
| *Physical exertion* | 762 | 0.82 (0.04) |  | 552 | 0.84 (0.05) |  | 0.02  (-0.09 to 0.13) | 0.716 |
| *Physical discomfort* | 762 | 1.45 (0.05) |  | 552 | 1.47 (0.06) |  | 0.01  (-0.11 to 0.13) | 0.848 |
| *Lack of motivation* | 762 | 1.18 (0.06) |  | 552 | 1.14 (0.06) |  | -0.04  (-0.18 to 0.09) | 0.515 |
| *Sleepiness* | 762 | 1.89 (0.06) |  | 552 | 1.86 (0.07) |  | -0.03  (-0.17 to 0.11) | 0.644 |
| ** Mean: Estimated margin mean, SE: Standard error, CI: confidence interval*  Values in **bold** indicate statistically significant results, with p < .005. | | | | | | | | |

| **Table S6.** Results from intention-to-treat analysis (adjusted model) on primary outcomes. | | | | | | | | | | |
| --- | --- | --- | --- | --- | --- | --- | --- | --- | --- | --- |
|  | **Shift schedule with abated quick returns** (intervention) | |  | **Shift schedule with quick returns**  (control) | |  |  | **Intervention effect**  **Adjusted model^a^** | | |
|  | No. | Mean* (SE) |  | No. | Mean* (SE) |  |  | Coefficient  (95% CI) | Cohen’s d | P-value |
| **Bergen Insomnia Scale** | | | | | | | | | | |
| Baseline | 647 | 15.46 (0.05) |  | 667 | 15.60 (0.06) |  |  | **1.53**  (0.24 to 2.81) | **-0.13** | **0.020** |
| Follow-up | 306 | 13.66 (0.45) |  | 246 | 15.32 (0.47) |  |  |  |  |  |
| **Epworth Sleepiness Scale** | | | | | | | | | | |
| Baseline | 647 | 8.33 (0.03) |  | 667 | 8.41 (0.04) |  |  | **0.78**  (0.18 to 1.37) | **-0.14** | **0.011** |
| Follow-up | 306 | 7.43 (0.20) |  | 246 | 8.28 (0.21) |  |  |  |  |  |
| **Revised Swedish Occupational Fatigue Inventory** | | | | | | | | |  | |
| *Lack of energy* |  |  |  |  |  |  |  |  |  |  |
| Baseline | 647 | 1.86 (0.01) |  | 667 | 1.89 (0.02) |  |  | 0.03  (-0.23 to 0.29) | -0.02 | 0.812 |
| Follow-up | 306 | 1.99 (0.10) |  | 246 | 2.06 (0.09) |  |  |  |  |  |
| *Physical exertion* |  |  |  |  |  |  |  |  |  |  |
| Baseline | 647 | 0.81 (0.01) |  | 667 | 0.83 (0.01) |  |  | 0.13  (-0.05 to 0.31) | -0.07 | 0.151 |
| Follow-up | 306 | 0.91 (0.05) |  | 246 | 1.05 (0.08) |  |  |  |  |  |
| *Physical discomfort* |  |  |  |  |  |  |  |  |  |  |
| Baseline | 647 | 1.43 (0.01) |  | 667 | 1.47 (0.02) |  |  | 0.14  (-0.06 to 0.34) | -0.08 | 0.163 |
| Follow-up | 306 | 1.40 (0.08) |  | 246 | 1.59 (0.06) |  |  |  |  |  |
| *Lack of motivation* |  |  |  |  |  |  |  |  |  |  |
| Baseline | 647 | 1.13 (0.02) |  | 667 | 1.17 (0.02) |  |  | 0.04  (-0.18 to 0.26) | -0.04 | 0.730 |
| Follow-up | 306 | 1.15 (0.08) |  | 246 | 1.27 (0.06) |  |  |  |  |  |
| *Sleepiness* |  |  |  |  |  |  |  |  |  |  |
| Baseline | 647 | 1.86 (0.01) |  | 667 | 1.88 (0.01) |  |  | 0.12  (-0.09 to 0.33) | -0.07 | 0.257 |
| Follow-up | 306 | 1.70 (0.7) |  | 246 | 1.84 (0.07) |  |  |  |  |  |
| ** Mean: Estimated margin mean, SE: Standard error, CI: confidence interval*  *^a^ Adjusted model: Crude model additionally adjusted for sex.*  Values in **bold** indicate statistically significant results, with p < .005. | | | | | | | | | | |
